# Autoimmunity: Are we asking the right question?

**DOI:** 10.3389/fimmu.2022.864633

**Published:** 2022-11-03

**Authors:** Polly Matzinger

**Affiliations:** Ghost Lab, Division of Intramural Research, National Institute of Allergy and Infectious Diseases, Bethesda, MD, United States

**Keywords:** autoimmunity, autoreactivity, T helper cells (Th cells), programmed cell death, danger model, tissue resident cells, tissue-based class control, pregnancy

## Abstract

For decades, the main question immunologists have asked about autoimmunity is “what causes a break in self-tolerance?” We have not found good answers to that question, and I believe we are still so ignorant because it’s the wrong question. Rather than a break in self-tolerance, I suggest that many autoimmune diseases might be due to defects in normal tissue physiology.

## Introduction

This essay is a stab at explaining autoimmune diseases starting with the idea that many (if not most) are caused by injury or other non-physiological events in a tissue (1), or by defects in the detection or handling of dying, injured or stressed cells (2, 3), rather than by defects in the immune system. It offers explanations for details of various diseases that have not been offered before, such as why few of us get autoimmune disease though most people carry autoreactive T and B cells; why the relationship between pregnancy and lupus is so complex; why vitiligo depigmentation occurs where it occurs; why multiple sclerosis (MS) brain lesions seem to be randomly distributed; how muscle cells in the thymus can lead to myasthenia; why some people missing blood clotting factors make inhibitory antibodies to the injected replacements while others don’t; why joints are particularly susceptible to inflammation; why women are more susceptible to some autoimmune diseases than men, and why this is not true of all autoimmune diseases; and why the frequency of autoimmune diseases seems to be rising faster than can be explained by changes in human genetics. Though the explanations that the danger model offers may certainly be wrong, the fact that a simple model of immunity can explain so many odd details, without adding special new situation-specific assumptions, suggests that it might be useful.

## Immunological responsibilities of tissues and organs

The danger model (1) suggests that the ultimate control of immunity lies with the bodily tissue cells, rather than either the adaptive or innate immune systems. It proposes that tissues have three immunological functions: 1) initiate immune responses; 2) induce tolerance and 3) set the local default effector class (4, 5).

### Initiating immune responses

Cells monitor their own health in many ways, using evolutionarily ancient detectors (once thought to be limited to APCs, such as TLRs, NLRs, RLRs, CLRs, CDSs, HSPs etc.) to detect misplaced or invading nucleic acids, membrane breaks, unfolded proteins and other cellular anomalies (6). When these detectors are activated, cells respond by generating antimicrobial peptides, initiating unfolded protein responses, undergoing programmed cell death, or producing/releasing other alarm signals (DNA, RNA, heat shock proteins, mitochondria, type 1 interferons, ATP, HMGB-1, etc.), generally known as DAMPs (7), alarm signals (2), or alarmins (8), that initiate repair processes, and/or activate local APCs and/or neutrophils.

### Inducing tolerance

Although T cells specific for many self-antigens are deleted in the thymus, such tolerance is not complete. There are tissue-specific antigens not expressed in the thymus (9), in spite of AIRE (10), and thus peripheral tolerance mechanisms must also exist.

A healthy tissue can induce T cell tolerance in at least two ways. First, it simply carries on its normal function and expresses its normal panoply of molecules without the release of alarm signals, and without co-stimulatory signals. Tissue-specific T cells are thus deleted (11) for lack of co-stimulation (12, 13) after they bind directly to a tissue cell, or to tissue antigens captured by resting APCs (11). Second, it expresses co-inhibitory signals (14), such as B7-H3 (15) or PD-L1 (AKA B7-H1) (16), that suppress destructive responses (14).

Some organs will be better at inducing tolerance than others. A newly hatched liver-specific T cell will soon traffic through the liver or its draining lymph nodes, and be deleted. A brain-specific T-cell, however, is in a different situation. Although there is some T-cell traffic through the brain, there isn’t much (17), and a new T cell might circulate for some time before encountering brain antigens. Thus, at any moment, there are likely to be more circulating self-reactive T cells specific for small and/or well barricaded organs (like brain or islets of Langerhans), than for large or easily accessed organs (like skin or liver).

In addition, tolerance to transient molecules will be difficult, especially those induced by conditions of damage/danger/infection. Examples could include the clipped and cleaved forms of complement and clotting factors, inflammatory cytokines, alarm signals and other danger-associated molecules.

Whenever tissue damage occurs, newly-born autoreactive T cells can become activated alongside T cells specific for the instigating agent. For example, a viral infection in the liver could stimulate anti-liver cytotoxic T cells along with the anti-virus T cells, because the activated APCs would present both viral and self antigens. Both sets of T cells would kill some liver cells before dying or returning to the draining node to become reactivated by APCs presenting their antigen. Along with the virus-specific T cells, the anti-liver T cells would continue to be activated until the infection is cleared. Though this looks like the beginning of an autoimmune disease, it is self-limiting for several reasons. First, activated APCs die after for 3-21 days (18, 19) and immune-induced cell death does not activate the new APCs that replace them (20). Thus, when the pathogen is cleared, activated APCs also disappear. Both the autoreactive and pathogen-reactive T cells will then die or become resting memory cells. Then, because the healed liver is large and ever-present, the resting autoreactive memory T cells will again encounter their antigen without co-stimulation and be deleted, while the anti-viral memory cells will continue to circulate.

Such transient autoreactivity may occur during many/most immune responses. It could account for the transient production of autoantibodies seen during some virus infections (21, 22). Overall, however, such transient autoreactive responses do not turn into autoimmune disease because the infected tissues return to health, and again become tolerogenic.

### Setting the local default effector class

Inflammatory immune responses can be lethal to local tissues. For example, a Th1 response (such as inflammatory cytokines, activated CTL and NK cells, macrophages making oxygen radicals, and/or complement-fixing antibodies) can destroy the eye (23), placenta (24), islets of Langerhans (25, 26), lung (27, 28) and likely many other tissues, whether the response is directed against self or a pathogen. I previously suggested that tissues may have mechanisms to control the effector class of a local immune response such that it doesn’t cause more tissue destruction than the tissue can tolerate (2) We and others have since uncovered a few of these mechanisms. First, tissues, or their resident lymphocytes (29), seem to educate their local APCs to induce certain classes of response. For example, the gut “trains” its APCs to induce Th2/Th3 responses (30) and to instruct T cells to home to the gut (31–34). The lung influences its macrophages in ways calculated to preserve lung function as best as possible (27, 28). The eye produces cytokines that suppress Th1 responses while enhancing IgA (23). Second, some tissues express inhibitory molecules that suppress one class of T helper more than another. Fas Ligand, for example, expressed by the eye and the testes, induces death of Th1 more easily than Th2 T-cells (35) and PD-L1 reduces IFN-γ secretion, especially by CD8 T-cells (36), and enhances production of IL-10 (37), as does B7-H3 (15, 38). The general idea that tissues influence the effector class is gaining traction (39).

## Autoreactivity vs autoimmunity

Before getting into autoimmune diseases, we should distinguish between “autoreactivity” and “autoimmunity”. They are not the same. Some autoreactive T cells are beneficial, such as NK1 T cells, MAIT cells, DECT cells, some γδ T cells, some αβ T cells (in the gut), and probably more that we haven’t discovered yet. They are often tissue-resident, specific for ligands upregulated by injury or stress, and promote healing and appropriate immune effector classes. The DECT cells in skin, for example, respond to stress-induced molecules by releasing skin-healing keratinocyte growth factor (40).

Most individuals also carry autoreactive T and B cells specific for intracellular or transient molecules (such as active forms of the clotting components (41), some cytokines, alarm signals etc.), and such antibodies have little effect on healthy tissue. When tissues are damaged, however, these B cells can become activated, and secrete “housekeeping” antibodies (42, 43) to clean up cellular debris (42, 43). Antibodies to alarm signals may be particularly important, as persistent alarm signals could maintain immune responses longer than needed. Indeed autoantibodies to type 1 interferons are found during SARS-CoV2 infections, and wane when the infections clear (22).

These are all useful self-reactive cells, and I will refer to them only rarely below.

## Autoimmunity: The basic assumptions

Many researchers consider autoimmune diseases to be due to defects in the immune system, for example, failures of self-tolerance, or “regulation”. Earlier, I suggested that some autoimmune diseases might be due to defects in cell death (1), and/or defects in cell scavenging (2), and here I add the view that they might be due to *any* defect in normal tissue physiology. Let’s see where this takes us.

Here are the basic assumptions:

1) We are never completely self-tolerant, though we are constantly *becoming* tolerant. As long as the thymus and bone marrow are putting out new T cells and B cells, there will be new circulating autoreactive lymphocytes.
2) Alarm signals from damaged tissues can initiate immune responses.
3) The wrong effector class can destroy a tissue, even in the absence of autoreactive T or B cells.^1^
4) The mere presence of an antigen does not initiate or maintain a response; it requires an activated APC. Because activated APCs have a finite lifespan, and new ones must be activated to maintain or reboot a response, immune responses are not self-perpetuating.

With these assumptions in mind, I find that autoimmune diseases fall into four categories, three of which are not due to defective immunity.

## Four different categories of autoimmune disease

### Category 1: An undetected infection in the target organ (e.g. Lyme disease)

Diseases in this category are not really autoimmune. They are infections, and the immune system is doing its job (clearing the pathogen). Unfortunately, it damages the infected tissue in the process. For example, a mouse dying of an undetected LCMV (44) or Theiler’s virus (45) infection, or a human with EBV activation in the brain (46) would be thought to have an MS-like autoimmune disease, but the responding lymphocytes are specific for the virus, not self.

Similarly, Lyme disease was originally diagnosed as juvenile rheumatoid arthritis (47). Once *Borrelia burgdorferi* was discovered, treatment changed from immune suppressants to antibiotics^2^. Chronic active hepatitis was also once thought to be autoimmune but has now been shown to be due to viral infection (50). In these cases, the immune system is attempting (but failing) to clear an infection and damaging the target organ in the process. Perhaps many other cases of so-called autoimmune diseases might actually be responses to undetected infectious agents. Do we yet know every bacterial, viral, fungal or other pathogen? As we become better at analyzing genomes, it might be worth looking at the target organs in other organ-specific “autoimmune” diseases for non-human genomes and devising methods to clear them.

### Category 2: Molecular mimicry (e.g. Rheumatic Fever)

This long-standing category is subtly but importantly different from category 1, in that it includes a truly autoreactive component. The poster child here is rheumatic fever, where a portion of the lymphocytes specific for *Streptococcus* also seem to recognize an antigen on the heart. Based on self-non-self assumptions, where antigen is the driver of immunity, it has long been thought that such a cross-reaction between a self-antigen and an environmental one can initiate a persistent anti-self-response, maintained by the continuous presence of the self-antigen. However, we now know that antigen presence is not enough. It takes an activated APC to initiate immunity, and activated APCs don’t last forever. Once a damaging pathogen has cleared, the response stops for lack of co-stimulation, and the ensuing memory cells once again become tolerized by the persisting self-antigen. This view fits with the results of medical practice in rheumatic fever. First, when the infection is cleared, the auto-anti-heart response also disappears (51). Second, tolerance eventually overcomes autoreactivity. Patients are often kept on penicillin for years to prevent the reoccurrence of the infection, because the secondary response to a second infection could cause even more damage to the heart. After a number of years, however, the antibiotic treatment can be stopped and, although the patients may have a subsequent strep infection, the autoreactivity does not recur - suggesting that the heart has had time to induce tolerance to itself.

Another, more recent example, is a subset of Lupus, where bacterial infections, with their concomitant nucleic acids, can trigger the anti-DNA response that is one of the prime characteristics of this disease (52).

Diseases in this category are likely to flare. Although rapid memory immune responses may clear recurring infections fast enough that their presence is undetected, each recurrence can induce a cross-reactive autoimmune flare. Alternatively, low-lying persistent cross-reactive pathogens could underly chronic autoimmune responses.

Although both categories above involve infections, category 1 has no autoimmune component, and the infection must therefore lie in the target organ. In category 2, however, antigenic mimicry from an infection in one organ can induce cross-reactive lymphocytes that cause destruction in distant organs. A form of autoimmune uveitis, for example, has recently been shown to be due to T cells activated by bacteria in the gut (53). There are likely to be more of these yet to be discovered.

### Category 3: Bad death and other glitches in normal cell physiology (e.g. Lupus)

This category is unique to the danger model. It has no foundation in Burnet’s self-non-self model (54, 55) or Janeway’s “infectious non-self” model (56, 57).

Every day, all over the body, cells undergo normal apoptotic programmed cell death. In some places, death is constant (e.g. thymus, skin, bone marrow and gut). In others, it occurs regularly (ovary and uterus), or sporadically (as in germinal centers). Mutations in genes governing these deaths, or in the normal scavenging of dying cells (3), could result in the release of alarm signals.

As described above, normal cells constantly monitor their own health. Upon sensing a non-physiological event, a cell responds by undergoing one form or another of programmed cell death (apoptosis, necroptosis, pyroptosis etc.), or by generating antimicrobial peptides, or secreting type 1 interferons, or a multitude of other alarm signals that can do double duty as APC activators and lead to an immune response. These are all complex processes, governed by a large number of genes. A defect in any one of those genes could lead to defects in the detection, death or scavenging processes, and to consequent immune responses. Thus, a disease like lupus could be caused by a defect in cell physiology, cell death, cell scavenging or cell processing. For example, lupus can arise from lack of DNAse (58), a duplication in TLR7 (59, 60), a mutation in a macrophage scavenger receptor (3) and many other mutations (61). In addition, environmental substances that change/damage normal cellular physiology could also lead to immune activation. It is not the immune response that is at fault here, but abnormal cellular physiology.

This might explain why lupus comes in so many forms. The American College of Rheumatology lists 11 criteria for lupus; a combination of any four is enough for a lupus diagnosis. Such enormous variation might be expected if the underlying cause can be any defect in the myriads of processes of normal cell physiology. Lupus is not alone here. Somatic mutations causing necrotic cell death may underly some autoimmune hemolytic anemias (62), and melanomas may induce vitiligo (63).

Livia Casciola-Rosen has similarly proposed that some autoimmune conditions are created by damage and regeneration in distant organs, suggesting that scleroderma is due to a combination of the ischemic-reperfusion injury common in Reynaud’s syndrome, combined with accumulation of metals (64); that autoimmune myositis is caused by an underlying cancer (65); and that statin use can trigger necrotizing myopathy (66, 67). She makes two important points. First, that many autoimmune diseases are caused by a combination of injury and antigenic change. Second, she suggests that the autoantibodies paint a picture of the antigenic nature of the underlying cause of the disease and that careful study of the particular subsets of antibody specificities can lead us to that underlying cause (64). Although Livia emphasizes the view that fragmentation or other changes in the structure of self antigens is important in the etiology of the autoimmune diseases, I think that this is not necessary. We don’t need to invoke antigenic changes as a way to break self tolerance, because, as delineated above, we are never quite tolerant. However, though I disagree with this particular aspect of Casciola-Rosen’s model, the idea that autoimmune diseases can be initiated and sustained by various forms of cellular damage and or growth (as in tumors) (68) is an important one.

A question often asked is “why is lupus primarily seen in females?” The danger model offers two potential explanations. The first is based on the fact that there are cell deaths that occur in female but not male bodies. Every month we pop an egg, and kill off, clean up, and regenerate an entire uterine lining. That is a lot of death, growth and cleaning up to do and a lot of potential mutations in death, growth and scavenging genes. It might also explain why some autoimmune flares are less frequent in pregnant women (in whom these death and clean-up processes are temporarily suspended), only to increase again after giving birth.

One therapy could be to give young female lupus patients hormones that prevent cycling and see if they remain in remission while on this regimen (diagnosis by treatment). Most rheumatologists won’t try this, thinking that long-term amenorrhea isn’t “normal”. However, monthly cycling isn’t normal either. We did not evolve to cycle continuously. Until birth control pills were created, women were generally either pregnant or lactating most of their reproductive lives. I think that a proportion of lupus patients would respond well to this treatment. Supporting this view are the findings that the incidence of lupus flares tend to go down with menopause (69, 70), with ovarian failure (71), and (often) with pregnancy (72), while they go up with irregular menses (73), and (somewhat) with hormone replacement therapy (74).

Pregnancy is particularly odd. Some lupus patients flare less during pregnancy. Others flare more, and in others, lupus appears during pregnancy and disappears afterwards (72). Let’s look at these, remembering that programmed cell death is an integral part of fetal development.

**1)** Fewer flares: The disease is likely due to cellular abnormalities in menstrual cycling processes. No cycling, no flares.
**2)** More/stronger flares: The disease is likely due to defects in processes that either increase during pregnancy (for example, breast development or other cellular responses to hormones) or to genetic variants shared by mother and fetus, resulting in a double dose during pregnancy.
**3)** Flares only during pregnancy: The fetus may express a mutation in cellular physiology [new, or paternally inherited (75)]. Suppose that a fetus carries a defective gene for cellular scavenging. During fetal development, cells dying by programmed cell death need to be scavenged. Defective scavenging could lead to both the release of alarm signals from the unscavenged cells, and to developmental abnormalities that lead to fetal death. In such cases, the mother’s lupus symptoms would stop when the fetus miscarries (a common feature of such pregnancies). If the mother and her partner carried synergizing defects, she might have been diagnosed with lupus before her pregnancy, with symptoms that exacerbate during pregnancy and return to their “normal” lupus state after delivery. Or, if the shared genetic mutations were recessive, the mother might only flare during a pregnancy with a homozygous child. Perhaps this is why some mothers do better when they change partners (76)?

Another possibility for the female bias in lupus (and some other autoimmune diseases) is environment (77). With some exceptions (e.g. farmers using agricultural chemicals), women in most western countries toxify themselves more than men do. They polish the floors, wash the clothes and dishes, wear makeup, dye their head hair and remove their body hair with creams or waxes (behaviors that tend to start after puberty), and such toxicity can lead to injured and stressed cells. This may be part of the reason that the frequency of autoimmune diseases is rising faster than can be explained by changes in human genetics. It’s not easy to avoid the toxins in our environment, from the chlorinated steam in our showers to the chemicals in our food, clothing, homes and gardens; however, for anyone with an autoimmune disease, it might be well worth trying.

### Category 4: The wrong effector class (e.g. Type 1 diabetes?)

This category is different from the previous three, in that it does not refer to how a response is initiated or maintained, but rather to the kind of response that ensues.

As described above, tissues communicate with the immune system to set effector classes that don’t destroy the tissue. Any defect in either end of that conversation – say, a mutation in *Fas/Fas-L* (35), or *PD1/PDL-1* (36, 37), or a change in intestinal epithelium that results in a drop in local TGF-β production (78) or a lack of some of the tissue-resident lymphocytes that secrete class-specific cytokines, like the liver-resident NK-T cells that secrete copious amounts of IL-4 (79, 80) - could lead to dysregulated inflammation that injures the tissue. This would be especially destructive to small organs and those that do not regenerate well.

Suppose, for example, that islets of Langerhans, like many other tissues, cannot withstand a strong Th1 response, and that the pancreas therefore normally “tunes” an immune response to a (theoretical) Th 4.7 class, thus preventing the destructive Th1 or Th17 type. Now suppose that some children have mutations in the relevant molecules involved in the “tuning” process, or suffer from environmental toxins that disable or change it. In these individuals, a coxsackie virus infection in the pancreas (81, 82) (category 1), or a defect in the physiological wave of apoptosis that occurs in the pancreas at about the time of weaning (83) (category 3), or, in fact, any immune response initiated in/near or targeting the islets, could lead to inflammatory islet destruction. Supporting this view is the finding that diabetes can be prevented in diabetes-prone mice by switching a local immune response to a Th2 class of response (25). The response still occurs and lymphocytes still infiltrate the islets, but the islets survive.

Similarly, the brain cannot withstand strong inflammatory responses. If the brain’s “tuning” processes are intact, responses to local virus infections (46) or activation of retroviruses in the brain (84), or an infection in the periphery that cross-reacts with a molecule in the brain, should normally result in a tuned/tailored effector class that doesn’t destroy brain cells. But, individuals with mutations in the brain/immune communication system, or perhaps a vitamin D deficiency (85) could generate destructive inflammatory responses that create the immune-mediated lesions seen in Multiple Sclerosis (MS). In the case of an infection in a peripheral organ that cross-reacts with a brain antigen, the effector class set by the infected peripheral organ could also generate effector T cells that traffic to the brain and produce the lesions^3^.

## Other questions

With these four categories in mind, let’s look at the so-far-unanswered questions from the introduction and offer up some Danger Model-based suggestions.

*Why do MS lesions seem to be randomly distributed?* Microglia in the brain, like APCs in other tissues, normally rest quietly. However, damage to a peripheral nerve can stimulate them to express MHC and co-stimulatory molecules (87) to recruit/activate T cells. Perhaps injury to any peripheral nerve can do the same. Thus, for example, injury to the retina (bright light?) might induce T-cell-mediated destruction in the visual cortex. Damage to the nose (drug use, infection, chlorinated shower steam?) could induce a lesion in the piriform or orbital frontal cortex. In fact, infection or damage to any peripheral part of the body could lead to a lesion in the connected area of the brain. Because there is a three- to seven-day delay in microglial activation, it would take a very detailed diary to make this link. However, it could be tested in some animal models.

*Why does vitiligo depigmentation occur where it occurs?* It appears that alarm signals not only initiate immune responses, they are also necessary for the vascular endothelium to allow T-cell extravasation into a tissue (88). Thus, the depigmentation occurs in places that incur damage – elbows, sun-exposed skin, wounded skin.

*Why do some people with hemophilia make inhibitory antibodies to replacement clotting factors whereas others don’t*? This is most likely a matter of timing. Replacement clotting factors are usually given when patients are bleeding (with all the concomitant alarm signals and activated APCs). However, when the replacements are given routinely, during times of no bleeding, no illness and no vaccines, the children tend not to generate inhibitory antibodies (89) and should eventually become tolerant.

*Thymic muscle cells and myasthenia?* Thymectomy cures many myesthenics, and histology of those thymi reveals the ectopic presence of muscle cells expressing acetylcholine receptors, and germinal centers containing B cells making anti-acetylcholine-receptor antibodies (90). I suggest that those ectopic muscle cells are stressed, and consequently express the alarm signals that lead to the immune response. Although newly developing thymocytes should become tolerant of these myocytes, mature T cells circulating through the medulla will become activated^4^. Removing the thymus removes the stressed myocytes, eliminates the source of the alarm signals and stops the response.

*Why are joints particularly susceptible to inflammation?* Joints are constantly damaged, as shown by the fact that hemophilia patients have fairly constant microbleeds in their joints. However, hemophilics don’t bleed more often than others; their bleeds are simply more noticeable. This suggests that most of us have (unnoticed) microbleeds in our joints, contributing to the arthritic changes that are common in elderly people. Thus, joint cells likely send alarm signals more often than other tissues, calling in T cells and other aspects of inflammatory responses. A supporting hint comes from the finding that people with rheumatic diseases who suffer a stroke and become unilaterally paralyzed often repair arthritic changes in the paralyzed limbs (92). Thus, a body given respite from damage and alarm signals will heal!

## Coda

A referee for this essay asked *Why is the frequency of Rheumatoid arthritis (RA) climbing?*

The short answer is “I don’t know”.

The somewhat longer answer, and one that can be applied to many other diseases, including asthma and allergy, is that each year, we humans pollute our air, water, food, clothing, dwellings (and dog toys) with more and more chemicals that don’t exist in nature, and which our bodies have not evolved to deal with. Although the idea that pollution contributes to disease is not new, there have been few cases where pollutants have been clearly linked to autoimmunity. However, a recent study following more than 80 thousand Italians showed a strong correlation between large particle (PM10) pollutants in the air and the incidence of RA, but not with psoriatic arthritis, lupus or MS (93). Here’s how the Danger model would put these data together. First, antibodies to citrullinated proteins are the classic characteristic of RA. Second, diesel exhaust and other pollutants can cause airway cells to citrullinate proteins, which changes a protein’s charge and can cause it to unfold (94). Third, the exposed hydrophobic portions of unfolded proteins can act as alarm signals to trigger immunity (7). Fourth, it is almost impossible to generate tolerance to transient molecules that are themselves alarm signals. Thus, the result of the increase in pollution is an alarm-triggered immune response to the molecules that the pollutant changes. Further, if the relevant citrullinated peptide(s) are presented by only a few MHC alleles, then only some of the people with long term exposure to a relevant pollutant will be affected.

But why would a response in the lungs cause a problem in joints? If, as mentioned above, active joints are places where damage routinely occurs, and where the vascular endothelium is consequently activated, then joints would be a place where immune cells would easily traffic. It would not be surprising, therefore, that a response initiated in the lungs (or other tissues) might also end up in the joints.

The “Air pollution/diesel fuel” argument, however, doesn’t answer the second part of the reviewer’s question, namely “*why is the incidence of RA going up while pollution is going down?*” First, while general pollution may be going down, individual pollutants are also going up. Air pollution measurements are made on only a small subset of molecules. A recent finding from Dr. Ian Myles (personal communication) suggests that we might want to focus on more defined targets. He found that the incidence of RA might not correlate with just the general level of pollution, but with one of three specific chemicals, the strongest fit being with aluminum oxide, a molecule that is everywhere around us. It is present in dental implants, prosthetics, bulletproof windows, sapphires and rubies, microchips, antiperspirants, cosmetics, toothpaste, paint, furnace heat shields and diesel engines. Aluminum oxide can act as either a weak base or a weak acid, depending on its environment. When in particles, it can be used as an abrasive because of its hardness (near to that of diamond). Given the evidence that hyper citrullination can occur after membrane breaks (95), one could wonder if either the chemical or physical properties of aluminum oxide could precipitate the breaks that start the disease.

Is this handwaving? Of course it is! But it’s rational handwaving and makes a couple testable predictions. It also leads to the view that focusing on specific pollutants might lead to better answers than taking pollution as a whole. We need to narrow it down to the exact chemicals if we hope to create a world that doesn’t make us sick.

So far, we have focused on things, like pollutants and mutations that, when present, contribute to autoimmunity. There is also something that, when removed, might also be important, namely electrons. We are electrical beings, and use charged radicals (usually oxygen or nitrogen radicals) in defense against pathogens. If not carefully controlled, the radicals can also cause strong cellular damage (96). As most organisms are constantly connected to the earth, a flux of electrons from the earth would quickly neutralize any radicals that escaped from the local immune synapses. However, humans (and sometimes our pets) are the only mammals not constantly connected to the earth. We tend (in the developed world) to wear rubber-soled shoes, sleep on beds and live in high rises or otherwise electrically insulated homes. Thus, most of the time, the earth’s electrons are not available to us. Does this matter? There is some recent evidence that re-connecting us with the earth has beneficial effects on cortisol production, pain, stress, sleep, inflammation, wound healing, mood etc^5^ (97, 98). Although much more research needs to be done in this nascent area, it suggests that we may have lost contact with a potent source of protection from cellular damage and inflammation.

## Summary

Long ago, Vaz and Carvallo, in an essay about autoimmunity, asked "Are we looking for answers, or for different questions?" (99). The danger model’s viewpoint on autoimmunity offers a change in the types of questions we should ask. Rather than the old “what broke tolerance?” we should ask 1) what initiates the response that causes the disease? 2) what maintains the response? and 3) what determines the effector class of the response? Perhaps a change in the questions we ask will result in a different and more useful set of answers.

Finally, many autoimmune diseases are likely to be due to heterogenous causes (various mutations, different environmental toxins, infections etc). Neither lupus, nor MS, for example, are single-cause diseases. Thus clinical trials where only a subset of patients respond to a particular treatment should not be automatically dismissed for lack of “overall” efficacy (100, 101). We should leap on the treatments that really help that subset, re-study them in expanded populations, and implement them if their efficacy in that subset repeats, rather than subjugating those people to a lifetime of disease from which they might be freed.

## Data availability statement

The original contributions presented in the study are included in the article/supplementary material. Further inquiries can be directed to the corresponding author.

## Author contributions

The author confirms being the sole contributor of this work and has approved it for publication.

## Acknowledgments

I thank Dr. Kaska Wloka for unstinting and generous assistance with this essay, Dr. Stefania Gallucci for suggestions on the manuscript, nq Dr. Elana Shaw for emphasizing the idea that tolerance to transient danger-associated molecules would be difficult to achieve, Dr Ian Myles for sharing his unpublished data, and ABF Sirius for inspiration. This work was supported by the Division of Intramural Research of the National Institute of Allergy and Infectious Diseases, NIH.

## Conflict of interest

The author declares that the research was conducted in the absence of any commercial or financial relationships that could be construed as a potential conflict of interest.

## Publisher’s note

All claims expressed in this article are solely those of the authors and do not necessarily represent those of their affiliated organizations, or those of the publisher, the editors and the reviewers. Any product that may be evaluated in this article, or claim that may be made by its manufacturer, is not guaranteed or endorsed by the publisher.

## Footnotes

1 I am often asked what I mean by “effector class”. Although the term “class” is most often used as a reference to antibody sub-classes, I use it in the broader sense of “all the cells and molecules” that, together, create a particular kind of immune response: e.g. Th1, 2, 3, 17, 23 and all the others we have not delineated yet. Each “class” is a set of T cells secreting certain cytokines and the effector arm that those cytokines regulate. For example, a Th1 response is made up of Th cells that make IFN-γ and TNF, plus a panoply of really destructive things that work together: CD8 killers, complement-fixing antibodies and the cells that use them, (e.g. activated NK cells that kill directly or by ADCC using complement-fixing antibodies, and activated macrophages that produce oxygen radicals and opsonize their targets using complement fixing antibodies), plus APCs that produce IL-12, which causes NK cells to make IFN-γ, which feeds back on the APCs to make more IL-12 and TNF and IL-6. This type of response is immensely destructive. It is the immune system’s agent orange! Tissues that regenerate easily (skin, liver etc.) can allow this response, but those that don’t (e.g. eye, placenta) have ways to communicate with cells of the immune system to swing the local response to a different, less destructive class: such as a Th2 (using IgG1 or IgE, plus the eosinophils and basophils that use these antibodies) or Th3 (producing TGF-β which signals B cells to make IgA). Tissue-resident lymphocytes, as well as the tissues themselves, are likely to be involved in this local level of class control. As we discover ever more tissue-resident T cells, we are likely to find even more combinations of helpers and effectors and their combined immune classes of response.

2 In those cases where symptoms continue after the infection seems cleared, it has been suggested that the infection led to self-perpetuating autoimmunity, yet many of these patients do better if the antibiotics are continued. My interpretation of this phenomenon is that either: 1) the Borrelia persist somewhere (despite their absence in blood and joints); 2) infection with Borrelia has led to a co-infection with other agents that persist; 3) the infection has led to cross reactions with other environmental agents. The latter is a common feature of some viral infections (48) and there is no reason to assume that it can’t happen with bacteria. Thus, if a patient does better while taking antibiotics and worse without them, they should be allowed to continue to take them. We don’t yet know enough about this disease, and those who insist that a clear joint sample is sufficient reason to stop antibiotic treatment (49) may be overconfident that we haven’t missed something.

3 A speculative MS story. Suppose that a form of MS is caused by an intestinal infection that cross-reacts with a brain peptide. Some people carry the cross-reactive peptide, others don’t. Most people that express the cross reactive peptide have no disease because they don’t have an MHC allele that presents it. Others might have the right MHC but not the cross reactive peptide. Rare individuals have both the cross reactive peptide and the presenting MHC, but still have no disease because an infection in the gut generally leads to IgA, which doesn’t cause destruction in the brain. But an even rarer set of individuals might have the right MHC/peptide combination AND an anomaly in the gut that leads to overproduction of inflammatory cytokines [akin to the overproduction of IL-15 in Celiac disease (86)] that leads to destructive T cells that traffic to the brain.

4 The thymus, like any other organ, needs protection. Thus, mature circulating T cells can be activated by APCs in the medulla to generate the relevant protective responses (91).

5 Is this why a day at the beach makes us feel good or why children who live on farms are less prone to autoimmunity, allergy and asthma than children in cities?
